# No impairment of maximal oxygen uptake in patients diagnosed with long COVID?

**DOI:** 10.1007/s00421-025-05713-5

**Published:** 2025-02-14

**Authors:** René Garbsch, Frank C. Mooren, Boris Schmitz

**Affiliations:** 1 Department of Rehabilitation Sciences, Faculty of Health, University of Witten/Herdecke, Witten, Germany; 2 DRV Clinic Königsfeld, Center for Medical Rehabilitation, Ennepetal, Germany

To the editor: In a recent issue of the *European Journal of Applied Physiology*, Berg et al. (2024) investigated the impact of long COVID on physical capacity by comparing maximal oxygen uptake, lung function, oxygen cost of walking, and performance in the 6 min walking test between patients and healthy, age- and sex-matched controls. The authors suggest that physical exercise capacity (assessed by cardiopulmonary exercise testing [CPET]) was comparable between patients and controls, and concluded that patients diagnosed with long COVID with a prior mild to moderate SARS-CoV-2 infection are not affected by reduced physical exercise capacity (Berg et al. 2024). While we read this article with great interest and appreciate the potential insights it provides into Post-COVID-19 sequelae, we believe that the authors’ interpretation of their findings and drawn conclusions warrant reconsideration, particularly in light of the high variability in long/post-COVID phenotypes.

Data from our group (Garbsch et al. 2024), and findings from earlier studies in the field (Ostrowska et al. 2023) consistently demonstrate significant impairments in cardiopulmonary fitness and physical performance among patients with long-term sequale after SARS-CoV-2 infection. Reported limitations in peak exercise capacity range from 17.2 ± 2.6 ml/min/kg (*n* = 97) to 18.0 ± 4.3 ml/min/kg (*n* = 198) equal to 73.6 ± 15.0 and 81.0 ± 16.9% of predicted reference, respectively (Garbsch et al. 2024; Ostrowska et al. 2023). While this is also mentioned in the introduction section, the authors report remarkably high maximum oxygen uptake of 40.0 ± 12.8 ml/min/kg corresponding to 103 ± 27% of predicted reference in 20 patients (44 ± 16 years, 45% female) recruited at a Norwegian rehabilitation center. Of note, the center itself correctly states on its homepage that many post-COVID patients report difficulty participating in daily activities as before and many describe worse physical function in the form of reduced endurance and muscle strength after prolonged periods of inactivity (https://treningsklinikken.no). It appears that precisely these patients were not included in the present study, which already only involved a relatively small number of cases (*n* = 20, lead symptoms including fatigue [*n* = 19], dyspnea [*n* = 9], and palpitations/ chest pain [*n *= 9]) and can thus not be considered representative. Since the authors did not apply an established score to grade the severity of the condition, the assessment of the disease severity as mild to moderate is also questionable. While the included patients are comparable to patients seen in our clinic in terms of time after acute infection, only one patient had been hospitalized during the acute infection, which appears not representative (Garbsch et al. 2024; Ostrowska et al. 2023). Of note, patients also exhibited unusually high 6-min walking performance at 606 ± 118 m compared to recent studies with 352 ± 75 m (Doehner et al. 2024). The description of physical activity levels suggests a population used to regular exercise training, corresponding to the finding that their performance level after the infection still reaches about 100% of the predicted value. The authors attribute their observations to certain nation-specific factors, suggesting that patients maintained their average weekly activity levels even during the period of lockdown preventing deconditioning and a corresponding reduction in VO_2max_. While the authors do not provide the date or time span the study has been conducted, the data provided using the IPAQ-SF still suggested a drop in activity level from before the acute phase of COVID-19 to the assessment of ~ 38% (3177 ± 2077 to 1981 ± 2254 MET minutes/week). However, we believe that based on the limited sample size and even considering the age- and sex-matched controls, the authors’ conclusion that long COVID patients are unaffected by impaired physical exercise capacity is erroneous and has the potential for general misinterpretation. Considering the large heterogeneity in this patient population, the authors should have concluded that a long COVID diagnosis may still be present in the absence of reduced physical fitness but based on the lead symptom of fatigue. Regretfully, the title chosen by the authors indicates that exercise impairment is not a lead symptom of long COVID in general. We feel that this lack of accuracy does not reflect the considerable disease burden of many long/post-COVID patients which is often followed by significant, diverse psychosocial challenges. Current evidence strongly suggests that the majority of long COVID patients suffer from a significant reduction in physical exercise capacity in combination with symptoms such as fatigue, dyspnea and cognitive impairment (Gutzeit et al. 2024) and studies in the field of rehabilitation are seeking to increase physical exercise capacity and limit restrictions in physical functioning in affected patients (Doehner et al. 2024). However, we agree that it remains uncertain to what extent these reductions in exercise capacity are directly attributable to the acute COVID-19 infection itself or result from deconditioning due to inactivity driven by post-COVID-specific symptoms.
